# Patient Demographic and Socioeconomic Factors Associated With Physical Therapy Utilization After Uncomplicated Meniscectomy

**DOI:** 10.5435/JAAOSGlobal-D-22-00135

**Published:** 2022-07-11

**Authors:** Michael R. Mercier, Anoop R. Galivanche, Anthony J. Wiggins, Joseph B. Kahan, William McLaughlin, Zachary J. Radford, Jonathan N. Grauer, Elizabeth C. Gardner

**Affiliations:** From the Department of Orthopaedics and Rehabilitation, Yale School of Medicine, New Haven, CT (Dr. Mercier, Dr. Galivanche, Dr. Kahan, Dr. McLaughlin, Dr. Radford, Dr. Grauer, and Dr. Gardner); the Division of Orthopaedics, Department of Surgery, University of Toronto, Toronto, ON (Dr. Mercier); and the Department of Orthopaedic Surgery (Dr. Galivanche and Dr. Wiggins), University of California, San Francisco School of Medicine, San Francisco, CA.

## Abstract

The extent to which physical therapy (PT) is used after meniscectomy is unknown. The objective of this study was to estimate the extent to which PT is implemented after meniscectomy and to identify factors associated with its utilization.

**Methods:**

The Mariner PearlDiver database was queried to identify patients who underwent uncomplicated meniscectomy. The number of PT visits for each patient was tabulated. Logistic regressions were used to compare demographic factors associated with no use of PT and use of nine or more PT visits.

**Results:**

In total, 92,291 patients met inclusion criteria. Of these patients, 72.21% did not use PT and 27.8% used 1 or more PT visits. Of the patients who used PT, 19.76% had 1 to 8 PT visits and 8.03% had 9 or more PT visits. Older age and noncommercial insurance types were associated with no PT use. Male sex, Medicaid, and Medicare were associated with markedly lower odds of increased PT utilization.

**Conclusion:**

PT is used in the minority of the time after meniscectomy. Among patients who do use PT, however, notable variation exists in the amount of PT visits used. Patient age, sex, insurance status, and geographic variables were independently associated with PT utilization.

Meniscal tears are common orthopaedic injuries that may be caused by trauma or degenerative changes in the knee joint. For symptomatic tears causing functional limitations or complex tears, arthroscopic partial meniscectomy (APM) is often done. APM is one of the most commonly conducted orthopaedic procedures; in 2014, it was estimated that over 500,000 meniscectomies were done.^1,2^

Despite being considered a minimally invasive procedure, prior research has demonstrated that patients undergoing APM may experience pain and swelling leading to loss of range of motion and altered function, decreased quadriceps muscle strength, and reduced knee-related quality of life.^3,4,5,6^ Prior research has demonstrated that physical therapy (PT) after APM is associated with improvement in patient-reported knee function and range of motion.^7^ Thus, PT is commonly used as a rehabilitative therapy after APM, and a variety of postoperative protocols exist. However, the extent to which PT is used after surgery is currently unknown, and no universal consensus exists.^8,9^

Prior research has demonstrated that several clinical and nonclinical factors may influence routine outpatient PT use. Power et al.^10^ noted that total joint arthroplasty patients were more likely to be referred for PT if they had lower baseline activity levels, were older, and had fewer comorbidity diagnoses. Among patients with spine disorders at a single spine center, patients were more likely to be referred to a PT if they were college-educated and less likely if they were receiving disability insurance payments, older, and male.^11^ In a large cohort of patients with a variety of musculoskeletal conditions, Carter et al. demonstrated that several nonclinical factors are associated with receiving PT services, such as having a college or advanced degree or residing in an urban area.^12^ However, previous studies examining PT use have involved patients with heterogenous orthopaedic conditions, many of which were not for postoperative indications. Furthermore, factors driving the wide variability and range of PT use across the postoperative rehabilitation period for common outpatient orthopaedic procedures have yet to be investigated.^13^

Thus, the goal of this study was twofold: (1) to estimate the relative utilization of PT after APM and (2) to identify and characterize factors independently associated with both PT nonuse and increased PT use after APM. Elucidation of such factors will help clinicians better understand underlying factors driving disparities in PT utilization.

## Methods

### Data Source

The PearlDiver Humana Patient Records Database (PearlDiver) is a large, commercially available administrative claims database.^13,14^ The Mariner data subset of PearlDiver represents 122 million distinct patients encompassing claims from 2010 through quarter 2 of 2018. In part owing to its large patient population incorporating private and government insurance claims data, PearlDiver has been extensively used within sports medicine research.^15,16,17,18,19^ Given the deidentified nature of the database, this study was deemed exempt from institutional review board approval.

### Inclusion and Exclusion Criteria

The Mariner subset of the PearlDiver Humana Database was queried for cases of APM using the Current Procedural Terminology (CPT) code 29880 (medial and lateral arthroscopic meniscectomy) or 29881 (medial or lateral arthroscopic meniscectomy). Patients who underwent additional orthopaedic procedures at the time of APM were excluded from analysis, with the exception of the following concurrent procedures: arthroscopic limited synovectomy (CPT code 29875), arthroscopic extensive synovectomy (CPT code 29876), separate compartment chondroplasty (CPT code 29877), and arthroscopic removal of loose body (CPT code 29874). In doing so, patents who were undergoing more extensive procedures (such as major knee ligament reconstruction) were excluded from the analysis.

Furthermore, to select patients with an uncomplicated postoperative course, patients were excluded from the analysis if they experienced any documented complication or return to the operating room within the 90-day postoperative period.

### Physical Therapy Utilization

Postoperative PT visits during the 90-day postoperative period were identified using the following CPT codes: 97001, 97002, 97003, 97010, 97014, 97032, 97033, 97035, 97110, 97112, 97140, and 97530. The total number of postoperative PT visits used per patient was tabulated to create a frequency distribution for the patient cohort.

The mean and SD of PT visits per patient were calculated. Patients were grouped into one of three groups depending on the number of PT visits used: 0 visits, 1 to 8 visits, and nine or more visits. Cutoff for the highest PT utilization bin was determined by one SD above the mean number of PT visits.

### Surgeon-level Patterns of Physical Therapy Utilization

The National Provider Identifier number was extracted from insurance claims when made available in the database. The proportion of patients with nine or more visits was tabulated per the National Provider Identifier number.

### Study Variables

Univariate statistical analyses were conducted to compare age, sex, geographic region, and payor type across the three aforementioned patient groups. Analysis of variance was used to compare means of ages, and the Pearson chi squared test was used to compare frequency differences in sex, geographic region, and payor type. To identify factors independently associated with both PT nonuse and increased PT use (nine or more visits), two separate binary logistic regression models were constructed. Covariates controlled for in each model included all variables examined on univariate analysis.

Statistical significance was set at α = 0.05, and 95% confidence intervals (CIs) were reported. All statistical analyses were conducted using the native PearlDiver statistical processing interface. .

## Results

### Study Sample

**Figure 1:**
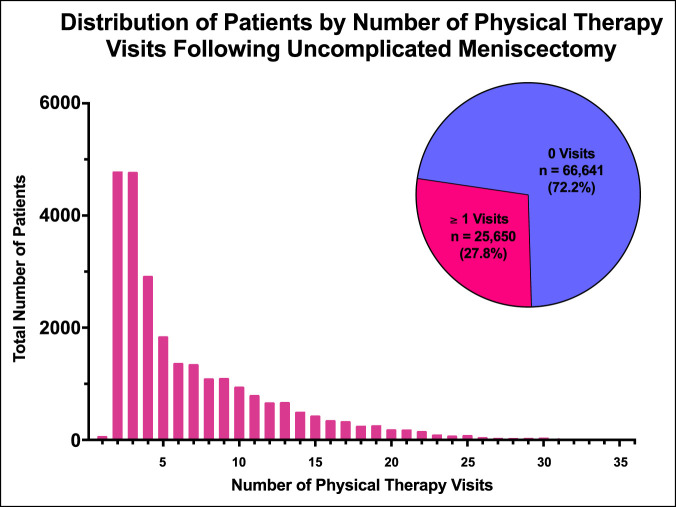
Diagram showing frequency distribution of patients using PT based on the number of visits used. The number of PT visits used ranged from 1 to 35. In total, 25,650 patients (27.8%) used one or more PT visit. PT = physical therapy

In total, 92,351 patients were identified who underwent APM and met inclusion/exclusion criteria within the PearlDiver Humana Mariner data set. Sixty-six thousand six hundred forty-one patients (72.2%) did not use PT, and 25,650 patients (27.8%) used one or more visits. Of the patients who did use PT, the mean number of visits was 3.45 and the SD was 2.81. The number of PT visits used ranged from 1 to 36 (Figure 1). Eighteen thousand two hundred thirty-six patients (19.76%) used 1 to 8 PT visits, and 7,414 (8.03%) used nine or more visits.

### Patient Demographic Factors

The mean age and SD of each PT utilization group were as follows: 54.93 ± 12.77 years (0 visits), 53.82 ± 12.86 years (1 to 8 visits), and 54.41 ± 12.76 (nine or more visits) (*P* < 0.001). Patients were increasingly female as PT utilization increased (0 visits: 51.57% female; 1 to 8 visits: 56.98%; nine or more visits: 60.13%, *P* < 0.001). Regarding geographic region, the Northeast demonstrated an increasing proportion of patients in the higher PT utilization groups (0 visits: 20.02%; 1 to 8 visits: 20.78%; nine or more visits: 33.73%, *P* < 0.001). For payor type, commercial insurance comprised a consistent increasing proportion of patients across PT utilization groups (0 visits: 79.48%; 1 to 8 visits: 88.23%; nine or more visits: 90.29%, *P* < 0.001). Conversely, Medicare saw a decreasing trend across PT groups (0 visits: 13.88%; 1 to 8 visits: 11.46%; nine or more visits: 10.97%, *P* < 0.001). Univariate analyses of patient demographic factors are summarized in Table 1.

**Table 1:** Demographics of Patients Undergoing Uncomplicated Meniscectomy Organized by the Number of Postoperative PT Visits

No. of PT Visits N = 92,291 (100%)	0 Visits N = 66,641 (72.21%)	1 to 8 Visits N = 18,236 (19.76%)	9 or More Visits N = 7,414 (8.03%)	*P* Value
Age (yr): Mean [SD]	54.93 [12.77]	53.82 [12.86]	54.41 [12.76]	**<0.001**
Sex				**<0.001**
Female	34,364 (51.57%)	10,391 (56.98%)	4,458 (60.13%)	
Male	32,277 (48.43%)	7,845 (43.02%)	2,956 (39.87%)	
Region				**<0.001**
Midwest	18,082 (27.13%)	6,134 (33.64%)	1,245 (16.79%)	
Northeast	13,343 (20.02%)	3,789 (20.78%)	2,501 (33.73%)	
South	25,104 (37.67%)	6,242 (34.23%)	2,765 (37.29%)	
West	10,033 (15.06%)	2,057 (11.28%)	895 (12.07%)	
Payor type				**<0.001**
Commercial	52,969 (79.48%)	16,090 (88.23%)	6,694 (90.29%)	
Government	1,333 (2.00%)	412 (2.26%)	171 (2.31%)	
Medicaid	2,469 (3.70%)	903 (4.95%)	183 (2.47%)	
Medicare	9,253 (13.88%)	2,090 (11.46%)	813 (10.97%)	

PT = physical therapy

Data in bold indicate statistical significance at *P* < 0.05.

### Multivariable Regression

On binary logistic regression analysis, PT nonuse was found to be positively associated with the following covariates: age (per decade, odds ratio [OR] = 1.04; 95% CI = 1.02 to 1.05; *P* < 0.001), male sex (OR = 1.32; 95% CI = 1.29 to 1.37; *P* < 0.001), the South (relative to the Midwest, OR = 1.13; 95% CI = 1.09 to 1.17; *P* < 0.001), the West (OR = 1.37; 95% CI = 1.31 to 1.44; *P* < 0.001), government insurance (compared with commercial insurance, OR = 1.16; 95% CI = 1.04 to 1.30; *P* = 0.006), Medicaid (OR = 1.32; 95% CI = 1.21 to 1.43; *P* < 0.001), and Medicare (OR = 1.48; 95% CI = 1.41 to 1.56; *P* < 0.001). Conversely, compared with the Midwest, the Northeast was found to be negatively associated with PT nonuse (OR = 0.87; 95% CI = 0.84 to 0.91; *P* < 0.001, Table 2).

**Table 2:** Factors Associated With No PT Utilization (0 Visits) After Uncomplicated Meniscectomy

N = 92,351 (100%)	Likelihood of Topbox Response		
	OR	95% CI	*P* Value
Age (per decade)	1.04	1.02 to 1.05	<0.001
Sex			
Female^a^	1.00	—	—
Male	1.32	1.29 to 1.37	<0.001
Region			
Midwest^a^	1.00	—	—
Northeast	0.87	0.84 to 0.91	<0.001
South	1.13	1.09 to 1.17	<0.001
West	1.37	1.31 to 1.44	<0.001
Payor type			
Commercial^a^	1.00	—	–
Government	1.16	1.04 to 1.30	0.006
Medicaid	1.32	1.21 to 1.43	<0.001
Medicare	1.48	1.41 to 1.56	<0.001

CI = confidence interval, OR = odds ratio, PT = physical therapy

a Reference category.

**Figure 2:**
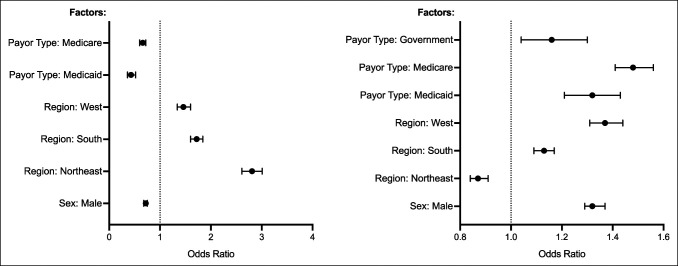
**A**, Forest plot depicting factors independently associated with nine or more postoperative PT visits (superuse). **B**, Forest plot depicting factors independently associated with no use of postoperative PT. PT = physical therapy

Increased PT utilization (nine or more visits) was found to be positively associated with the following geographic regions: the Northeast (compared with the Northwest, OR = 2.81; 95% CI = 2.61 to 3.01; *P* < 0.001), the South, (OR = 2.81; 95% CI = 1.72; *P* < 0.001), and the West (OR = 1.46; 95% CI = 1.34 to 1.60; *P* < 0.001). The following covariates were found to be negatively associated with increased PT utilization: male sex (OR = 0.72; 95% CI = 0.68 to 0.75; *P* < 0.001), Medicaid (OR = 0.43; 95% CI = 0.36 to 0.52; *P* < 0.001), and Medicare (OR = 0.66; 95% CI = 0.60 to 0.72; *P* < 0.001). These results are summarized in Table 3, and statistically significant findings of both regressions are presented in Figure 2.

**Table 3:** Factors Associated With Increased PT Utilization (9 or More Visits) AFter Uncomplicated Meniscectomy

N = 92,351 (100%)	Likelihood of Topbox Response		
	OR	95% CI	*P* Value
Age (per decade)	0.99	0.97 to 1.01	0.310
Sex			
Female^a^	1.00	—	—
Male	0.72	0.68 to 0.75	<0.001
Region			
Midwest^a^	1.00	—	—
Northeast	2.81	2.61 to 3.01	<0.001
South	1.72	1.60 to 1.84	<0.001
West	1.46	1.34 to 1.60	<0.001
Payor type			
Commercial^a^	1.00	—	—
Government	0.92	0.77 to 1.10	0.349
Medicaid	0.43	0.36 to 0.52	<0.001
Medicare	0.66	0.60 to 0.72	<0.001

CI = confidence interval, OR = odds ratio, PT = physical therapy

a Reference category.

## Discussion

Meniscal tears are among the most common injuries to the knee and occur across a wide range of patient ages and demographics. Although several trials have compared the clinical efficacy of APM versus PT, little research has focused on the role of PT after APM in functional rehabilitation.^20,21,22,23^ Consequently, patterns of postoperative PT utilization after APM are highly variable and may be influenced by nonclinical factors. Thus, this study sought to characterize the extent to which PT is used after APM and to further elucidate factors that may influence its utilization. Here, we use a large, national insurance claims database to demonstrate that a minority of patients used any form of PT after APM. Several factors were shown to be associated with increased PT utilization, including the treating orthopaedic surgeon, age, sex, geographic region, and payor type.

This study demonstrate that 27.8% of the patients used one or more PT visits after APM, with 19.76% of the patients using 1 to 8 visits and 8.03% using nine or more visits. To the authors' knowledge, this study is the first of its kind to approximate the degree of PT utilization for APM. This rate is concordant with prior research conducted by Wagner et al.,^24^ who reported a 31% PT utilization rate among patients undergoing total shoulder arthroplasty in the PearlDiver Humana private insurance database. However, Wagner et al.^24^ also noted notable variation in the utilization rate between insurance groups, with a 56% utilization rate in an analogous Medicare population. In total knee arthroplasty surgery, prior research has demonstrated considerable variability in timing, utilization, and exercise content of PT after surgery.^25^ Given the current study's low overall PT utilization rate with notable variability, future research should work to identify and standardize optimal timing and the amount of PT.

In addition to the notable variability demonstrated in patient-level PT utilization, a wide spectrum of physician-related PT prescription patterns was also observed. There are several potential factors that may be contributing to this observation. This study, in addition to prior research, has demonstrated a strong association between insurance type and PT utilization in orthopaedics.^24,26^ Thus, providers may systematically adopt particular PT prescription patterns based on the predominant insurance type(s) that they routinely accept and see. In addition, prior research has demonstrated that surgeon-level PT utilization patterns may be influenced by a provider's own financial stake in PT services. In particular, Mitchell et al. showed that physical therapists not involved with physician-owned clinics saw patients for fewer visits.^27^ Physicians typically function within a larger organization, and thus, organization factors may influence their referral treatment patterns.^11,28^

Increasing patient age was independently associated with PT nonuse. Regarding age, these findings align with Freburger et al. who showed that patients with spine disorders were less likely to be referred to a physical therapist if they were 50 years or older.^11^ A similar study demonstrated that variations in PT use, explained by factors other than need, may suggest systematic underuse of PT by community-based older people.^29^ One proposed reason for lower PT use among older patients may be logistic barriers, such as assistance with transportation to and from PT visits. Elderly patients who may rely on others for transportation may need to forgo less crucial health services such as PT.

Male sex was found to be positively associated with PT nonuse and negatively associated with increased PT utilization. This finding is concordant with an aforementioned multicenter study of spine patients, which showed that male patients were less likely to be referred for PT.^11^ Referral patterns based on patient sex may be due to baseline differences in willingness to engage with PT services or acknowledgment of its potential benefit.

Notable variation was observed in PT utilization across geographic regions on multivariable analysis. In particular, the Northeast demonstrated lower odds of PT nonuse and higher odds of increased PT use. This is concordant with a multicenter spine study, which demonstrated higher PT utilization in the Northeast for patients with back and neck pain.^30^ Variations in healthcare use based on the geographic region have been noted, and differences may be attributed to differences in physician practice style.^31,32^ Furthermore, differential amounts of PT utilization may be driven by an uneven distribution of physical therapists. PT supply has been shown to be positively associated with PT use and the amount of PT received.^29,33,34^ Interestingly, the South and West both demonstrated higher odds of PT nonuse and higher odds of increased PT use (nine or more visits) compared with the Midwest. This finding suggests a potential bimodal phenomenon of PT utilization among patients. Future research should attempt to identify drivers on both ends of the spectrum (PT nonuse and increased use) in these geographic regions.

Perhaps the most compelling factor associated with PT utilization was payor type. Compared with commercial insurance, Medicaid and Medicare demonstrated substantially higher odds of nonuse and correspondingly decreased odds of increased utilization. Noncommercial insurances are frequently cited as barriers to access to rehabilitation services in sports medicine.^35,36,37,38,39^ Like many orthopaedic procedures in sports medicine, the timing and appropriate amount of PT in APM is unclear. Given this clinical uncertainty, variability in PT utilization is high and presumably influenced by permissive factors such as favorable commercial insurance. Treating surgeons should be cognizant of the potential barriers insurance may pose to their patients in access to PT and advocate for noncommercially insured patients who would benefit most from it.

There are several limitations under which this study should be considered, many of which are intrinsic to the PearlDiver database. Notably, the population of PearlDiver is not randomly sampled and thus not a true nationally representative estimate.^40^ Data within the PearlDiver database are predominantly of patients 65 years and older, and sampling favors the Southern United States.^14^ However, the database contains records on nearly 122 million individuals and is thought to ultimately represent a broad spectrum of patients. In addition, given that PearlDiver data are derived from insurance claims, access to more granular data such as comorbidities is difficult to reliably estimate.^40,41^ In a similar vein, additional sources of PT, such as athletic team trainers and home exercise programs, may not be accurately captured if it was not documented by a claim. Finally, we are unable to ascertain the specific reason (either clinical or nonclinical) why a patient ultimately decided to not pursue PT or, conversely, continued to go an increased amount of times. Clinically meaningful end points after APM are not available; thus, objective criteria to determine a patient's need to start or stop PT after surgery cannot be assessed.

Nevertheless, there are several strengths of this study that should be appreciated. Utilization of PearlDiver to study APM allows for an unprecedented sample size of over 92,000 patients. APM is reliably captured by distinct and specific CPT codes, as are the PT services that were tracked. CPT codes are known to be coded with high fidelity in insurance claims data, allowing for identification of surgeries and PT with relatively high specificity.

This study demonstrates that a minority of patients use PT after APM. Among patients who do use PT, a wide spectrum of utilization was noted. Variations in PT use were influenced by the specific provider, and several factors such as patient age, sex, geographic region, and payor type were markedly associated with PT utilization. These findings are important for providers because they highlight potential underuse or overuse of rehabilitation services depending on factors other than a patient's clinical need. Future research should work to determine the optimal amount, extent, and timing of PT services after APM to better standardize treatment.
